# Early-Onset Parkinson’s Disease: Unique Features and Management Approaches

**DOI:** 10.1007/s11910-025-01470-2

**Published:** 2025-12-18

**Authors:** Jordan Hickman, Andrew Tsai, Michelle Fullard, Michael Korsmo, Emily Forbes, Sana Aslam, Alexander J. Baumgartner, Jeanne S. Feuerstein, Ece Bayram

**Affiliations:** 1 University of Colorado Anschutz Medical Campus, Medical Scientist Training Program, Aurora, CO USA; 2 University of California Los Angeles, The UCLA Institute for Society and Genetics, Los Angeles, CA USA; 3 Department of Neurosurgery, University of Colorado, Aurora, CO USA; 4 Department of Neurology, Rocky Mountain Regional VA Medical Center, Aurora, CO USA; 5 Movement Disorders Center, Department of Neurology, University of Colorado Anschutz, 12469 E 19th Ave, Aurora, CO 80045 USA

**Keywords:** Early onset parkinson disease, Clinical features, Risk factors, Diagnosis, Biomarker, Treatment

## Abstract

**Purpose of Review:**

To highlight the unique clinical features, risk factors, and management strategies associated with early-onset Parkinson’s disease (EOPD), and contrast these with late-onset Parkinson’s disease (LOPD). We outline how these differences influence diagnostic and therapeutic approaches and identify key knowledge gaps critical to improving clinical care.

**Recent Findings:**

Compared to LOPD, EOPD (onset age 21-50) has a higher prevalence of monogenic risk factors, focal dystonia, depression, anxiety; slower motor progression; lower rates of cognitive decline; higher risk for delayed diagnosis. Treatment is complicated by earlier and more frequent dyskinesias, motor fluctuations, and unique considerations such as pregnancy and career impact.

**Summary:**

Risk factors, clinical presentation, progression, and management needs of EOPD can differ from LOPD. Despite advances in characterizing and diagnosing EOPD, most research remains focused on LOPD. There is a critical need to tailor research and clinical trials to address the distinct needs of people with EOPD.

## Introduction

Parkinson’s disease (PD) is the fastest-growing neurological disorder affecting over 11 million people worldwide [1]. It is characterized by bradykinesia, rigidity, tremors, and a wide range of additional motor and non-motor symptoms, with underlying dopaminergic loss [2]. Early-onset Parkinson’s disease (EOPD) is defined as PD with onset between ages 21 and 50 [3]. The International Parkinson and Movement Disorders Society (MDS) Task Force recommends using the term *“early-onset”* over *“young-onset”* to avoid age-related stigmatization, and an upper age cutoff of 50 aligns with majority of studies in the literature and the average age of menopause that likely influences PD risk [3]. EOPD accounts for 5–14% of PD, with many features differing from late-onset PD (LOPD) [4].

The global age-standardized prevalence of EOPD is estimated at 10.2, with an incidence of 1.3 per 100,000 people. Rates are higher for men and peak in the 35–39 age range [5]. The incidence and burden have risen since the 1990 s and are projected to continue increasing through 2035 [6]. These trends, coupled with the onset during individuals’ most professionally and socially engaged years, underscore the need for improved early diagnosis and effective treatments. In this review, we summarize the clinical features, diagnostic considerations, risk factors, and management strategies for EOPD compared to LOPD, to guide current practice and future research based on recent studies.

## Clinical Profile

The initial motor presentation in over half of people with EOPD is an asymmetric, akinetic-rigid syndrome; approximately one-third present with a tremor-dominant form, and a smaller proportion with a mixed phenotype [7]. At onset and during early stages, gait dysfunction and postural instability are typically absent and remain minimal for at least five years post-diagnosis, while more than half of those with LOPD can experience gait dysfunction within the first five years post-diagnosis [8]. Dystonia, while not a cardinal motor feature, occurs earlier and more frequently in EOPD than in LOPD [9].

Compared to LOPD, the overall prevalence of non-motor symptoms is lower in EOPD and the most reported symptoms in both groups are depression/anxiety (57% of LOPD vs. 48% of EOPD), urinary symptoms (51% of LOPD vs. 44% of EOPD), and sexual symptoms (52% of LOPD vs. 40% of EOPD) [10]. Depression can be more prevalent in EOPD than LOPD and it contributes significantly to reduced quality of life [3, 11]. Cognitive function is generally better preserved, with slower decline and lower incidence of dementia [12, 13]. Genetics can impact the risk level for cognitive decline; *PRKN* is associated with relatively lower risk of cognitive decline and dementia [14]. Restless legs syndrome and excessive sweating are more frequently reported in EOPD than LOPD, with the latter potentially linked to the higher incidence of levodopa-induced dyskinesias and motor fluctuations. Impulse control disorders and dopamine dysregulation syndrome also occur more often in EOPD, especially among men [15].

While disease progression is slower in EOPD than in LOPD, treatment-related motor complications are more common. The Hoehn and Yahr (H&Y) stage increases by approximately one point within the first decade of EOPD, whereas a similar increase occurs within two years for LOPD [7, 16]. Motor fluctuations and levodopa-induced dyskinesias typically emerge within 5–6 years of onset in PD but are more frequent in EOPD than LOPD, affecting approximately 80% and 60% in EOPD [7], respectively, versus ~ 54% and 14% in LOPD [17].

Survival also differs for EOPD and LOPD. EOPD is associated with a longer disease course with 25–32 years from symptom onset to death, compared to 7–14 years in LOPD [3, 18, 19]. This difference is largely attributable to an earlier age at onset. Cognitive decline remains a key factor associated with shorter survival in both groups.

### Effects on Daily Life

EOPD affects people during critical periods of career development, family responsibility, and social role establishment, creating unique challenges. Compared to LOPD, unemployment due to disability and early retirement rates are higher in EOPD [20]. Coupled with longer disease duration, these factors contribute to a greater financial burden over time. Quality of life is significantly lower in EOPD than LOPD, potentially driven by higher rates of depression and greater perceived stigma [21].

People with EOPD report experiences of stigma, shame, and grief related to both current and anticipated losses [22]. Identified unmet needs include tailored psychosocial support such as peer support, accessible information about disease progression, and targeted help with mental health and employment [23].

### Women with EOPD

Women with EOPD face unique, gender-specific issues that complicate symptom management and quality of life. Hormonal fluctuations related to menstruation and pregnancy can affect symptom severity and treatment response. Symptoms often worsen and medication effectiveness decreases during menstruation, and impaired dexterity may complicate menstrual hygiene [24–26].

There is no current evidence that PD impairs fertility. Carbidopa/levodopa is generally considered the safest pharmacologic option during pregnancy, with no evidence of major fetal abnormalities [27], though safety data remain limited. In a small study, nearly half of the participants reported symptom worsening during pregnancy (primarily following medication changes), while the other half noted no change, and one reported an improvement in symptoms [28].

## Risk Factors

Risk factors for EOPD include monogenic, polygenic, acquired and environmental factors, as well as interaction between genetic susceptibility and environmental exposures. Genetic factors are more common for EOPD than LOPD. Among the most common causes of monogenic EOPD are autosomal recessive mutations in *PRKN*, which, along with *PINK1* are critical to mitophagy, and assist in the removal of damaged mitochondria [29, 30]. Mutations in *DJ-1*, which accounts for ~ 1% of recessive EOPD, are also implicated in mitochondrial dysfunction among other processes [31]. Autosomal dominantly inherited mutations in *SNCA*, which encodes α-synuclein, cause EOPD of variable severity, although individuals with duplication and triplications of this gene appear to have a dose-dependent increase in severity and earlier age of onset [32].

In the North American PDGENEration cohort, reportable variants were identified in 18.4% of people with EOPD; notably higher than the overall cohort with reportable variants identified in 12.9% across all participants [33]. For individual genetic variant groups; 79% of the people with biallelic variants in *PRKN*, 21% of the people with a *GBA1* variant, and 9% of the people with *LRRK2* variants had EOPD. Mean age of onset for the *PRKN* group was 38.6 years, and for the *GBA* group was 58.6 years. In this cohort, causative variants in *SNCA*,* PINK1*, and *DJ-1* were rare, and subgroup analysis in EOPD was not performed.

Understanding monogenic drivers of EOPD in diverse populations is integral to understanding the full landscape of genetic architecture. The Latin American Research Consortium on the Genetics of PD (LARGE-PD) cohort found 5.6% of people with EOPD carried a copy number variant in *PRKN* [34]. For people with EOPD in India, 7% had a genetic causative variant with *PRKN* as a major driver. *SNCA* was also reported, in line with findings from European-ancestry cohorts. Other Indian cohorts also noted *GBA* at high frequency [35], and *DJ-1* for ~ 5% of EOPD [36]. A study in China found 120 causative variants in 82 (11.6%) of 704 individuals with EOPD including *PRKN* (5.1%), *LRRK2* (2%), *PINK1* (0.7%), and *SNCA* (0.5%). In addition, 7.2% carried a rare *GBA* variant. The study also noted three common pathogenic variants, p.A53V in *SNCA*, p.G284R in *PRKN* and p.P53Afs*38 in *CHCHD2*, which have been so far reported exclusively in people with Asian ancestry [37]. The International PD Genomics Consortium Africa demonstrated a novel *GBA* variant (rs3115534-G), which had not been previously noted in non-African or non-African admixed populations [38, 39]. Although EOPD was not specifically evaluated in this study, this variant affected age at onset, with onset of PD three years earlier per risk allele [39].

Polygenic risk scores (PRS) have increasingly been studied to determine the cumulative impact of common variants on disease risk. Although there are limited studies on PRS specifically in EOPD, several PRS to date have found associations with age of onset [40–43].

Acquired risks and environmental exposures interact with genetic susceptibility to influence both risk and age of onset; however, few studies have assessed environmental risk factors specifically in EOPD (Table 1). Ultimately, the gene-environment interaction likely plays a significant role in the development and age of onset of PD, with several studies assessing the influence of the interaction between PRS and environmental factors [42, 55].

**Table 1:** Acquired risk and protective factors for Parkinson’s disease (PD) with potential implications for early-onset PD (EOPD)

Category	Exposure	Description
Lifestyle Factors	Exercise	Exercise is a protective factor for development of PD, with a pooled hazard ratio of 0.66 when comparing the highest to lowest physical activity groups [44]. Limited data in younger people with PD indicate exercise is a protective factor [45].
	Diet	Studies in EOPD are limited; observational studies in PD indicate the Mediterranean diet may slow disease onset and progression [46, 47].
	Cigarette smoking	Smoking has a protective association with PD, with a stronger association among people with younger age of onset [48].
	Coffee and caffeine	Limited data on EOPD, however, there is a suggested dose-dependent response [49].
Trauma	Head injury	Head injury increases EOPD risk [45]. Unclear mechanism; neuroinflammation may play a role.
Toxicants	Pesticides	Pesticides and related exposures (rural living, farming, well-water exposure), although not studied in EOPD, indicate a dose-dependent response with a 5% increased risk for 5 years of exposure and 11% increased risk for 10 years of exposure [50, 51]. Well water was specifically evaluated as a risk factor in younger people with PD [45].
	MPTP	Chemically similar to pesticides, causing substantial nigra degeneration, originally reported in younger people [52].
	Trichloroethylene (TCE) and volatile solvents	Animal studies demonstrate a dose-dependent association between TCE and loss of dopaminergic neurons, and a larger study among military service members suggested a link between the risk of PD and exposure to water contaminated with volatile solvents [53, 54]. There is limited data on specific risk for EOPD.

## Diagnosis

The diagnostic criteria for EOPD are identical to LOPD. Diagnosis is clinical, requiring parkinsonism – classically bradykinesia and either rest tremor, rigidity, or both – and the exclusion of alternative causes [2]. People with EOPD have a higher risk for delayed diagnosis than LOPD, often due to assumptions that symptoms before the age of 50 stem from more common, non-degenerative causes like musculoskeletal injuries or idiopathic focal dystonia [56].

**Fig. 1:**
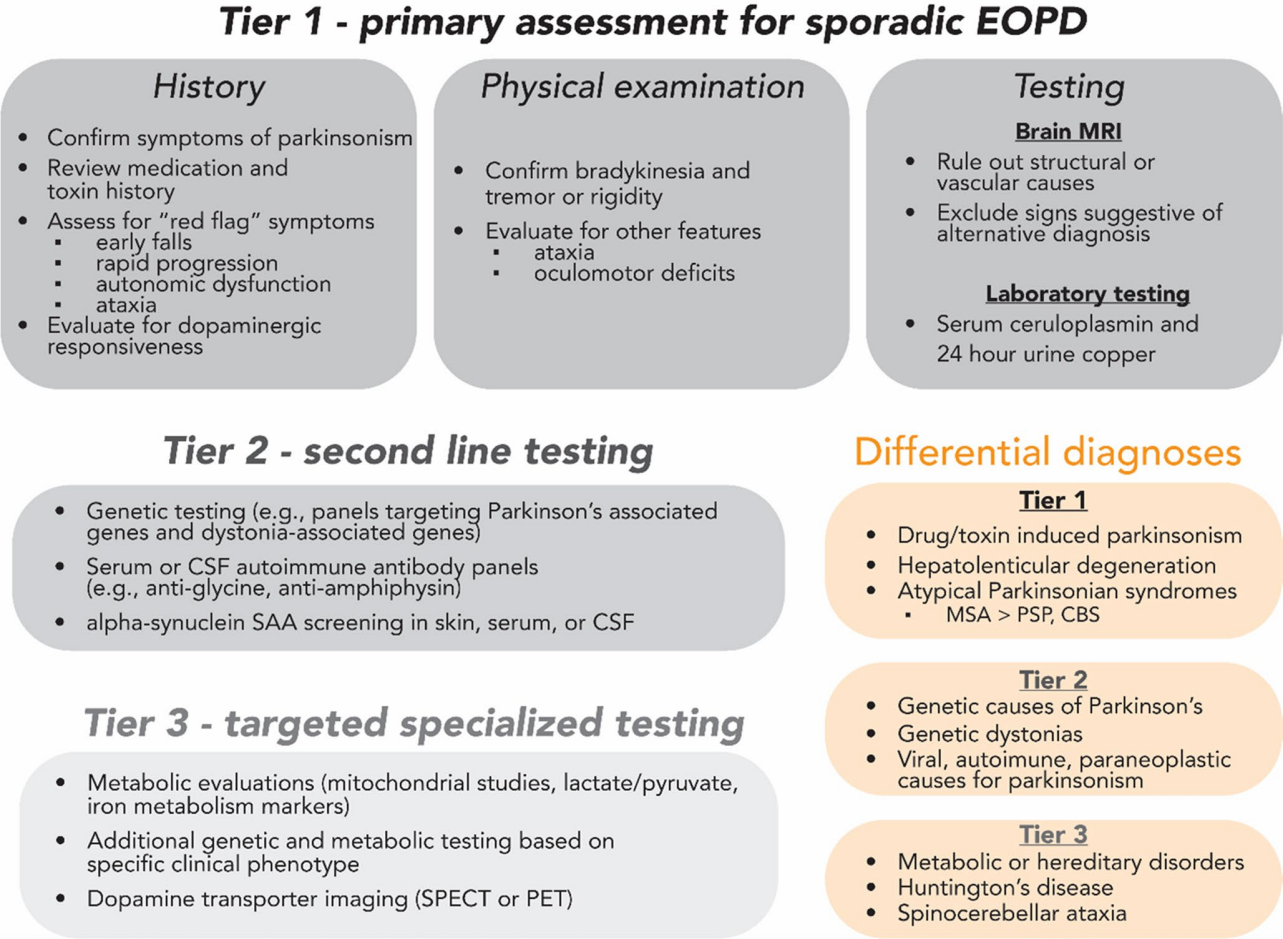
The Tier 1 assessment, adapted from [58], aligns with the proposed diagnostic approach by the MDS EOPD Study Group. Expanded testing should be done (Tier 2 and Tier 3) when accessible and clinically relevant. Acronyms: MRI: magnetic resonance imaging, MSA: multiple system atrophy, PSP: progressive supranuclear palsy, CBS: corticobasal syndrome, SAA: seed amplification assay, CSF: cerebrospinal fluid, SPECT: single-photon emission computed tomography, PET: positron emission tomography

A tiered approach will be helpful for the differential diagnosis (Fig. 1). Key categories for differential diagnosis include secondary causes of parkinsonism (drugs, exposures, etc.), hereditary or genetic movement disorders, and other synucleinopathies (i.e., multiple system atrophy) [57]. History and physical exam should be conducted to confirm parkinsonism, medication and toxin history, response to dopaminergic therapy, lack/presence of red flag symptoms (i.e., early falls, rapid progression, autonomic dysfunction, ataxia, oculomotor deficits). Neuroimaging should be routinely performed to exclude structural or vascular causes even in younger people [57]. The MDS EOPD Study Group recommends prioritizing brain MRI and laboratory investigation for hepatolenticular degeneration (Wilson’s disease) to initiate prompt treatment, especially if no additional findings for secondary or genetic parkinsonism are found on history and examination (Fig. 1) [58].

### Drug- and Toxin-Induced Parkinsonism

Drug-induced parkinsonism, the most common secondary cause, is typically linked to dopamine-blocking medications (antipsychotics and antiemetics), certain anti-epileptics, calcium channel blockers, and mood stabilizers. Symptoms usually resolve within six months after discontinuing the offending agent [59, 60]. Toxin exposures (e.g., manganese, carbon monoxide, pesticides, and MPTP) can cause parkinsonism and are associated with elevated risk of developing PD [45, 61].

### Other Secondary Causes

Infectious, autoimmune, and paraneoplastic causes, though rare, should be considered, especially in younger people [62].

### Genetic and Metabolic Disorders

Several genetic or metabolic disorders can cause parkinsonism in younger people including hepatolenticular degeneration (Wilson’s disease) [63], dopa-responsive dystonias (e.g., tyrosine hydroxylase deficiency) [64], Huntington’s disease [65], spinocerebellar ataxia [66], familial calcification syndromes, mitochondrial disorders, neurodegeneration with brain iron accumulations (NBIAs) [67], or leukodystropies [57]. Genetic testing and targeted laboratory tests are often warranted.

### Atypical Parkinsonism

Atypical Parkinsonian disorders, specifically progressive supranuclear palsy [68] and corticobasal syndrome [69], typically occur in older populations and are unlikely in younger people. Multiple system atrophy can present at an earlier age; it may be distinguished by lack of response to levodopa, characteristic imaging findings (putaminal rim sign, hot cross bun), prominent autonomic dysfunction or ataxia [57, 70].

### Biomarkers

Biomarkers to support PD diagnosis and staging are a prominent area of research and typically include biofluids, tissue-based sampling, and imaging [71]. The most extensively validated marker is pathological alpha-synuclein, the core protein in Lewy pathology. While markers such as glial fibrillary acidic protein, neurofilament light chain, and tau have shown some promise in cerebrospinal fluid (CSF) and serum, they generally lack disease specificity in isolation [71, 72]. Seed amplification assays (SAA) for misfolded alpha-synuclein have substantially improved diagnostic sensitivity and specificity. Notably, these assays can be applied to CSF, skin, gastric mucosa, and serum [73]. SAA can detect pathological alpha-synuclein in CSF with 95–99% sensitivity among LOPD cases with olfactory deficit. However, sensitivity of ~ 67% in *LRRK2*-associated PD [74], and reports showing no seeding with *PRKN* and *PINK1-*associated PD in small cohorts [75] underscore biological heterogeneity. EOPD-specific validation studies are lacking. Given the higher prevalence of genetic variants in EOPD, current alpha-synuclein biomarkers may not capture the full spectrum of disease. Differences in non-synuclein biomarkers between EOPD and LOPD have been reported in the literature; younger people with PD often have lower CSF tau and amyloid levels, likely reflecting a less co-pathology burden [76, 77].

Structural brain MRI is typically unremarkable in PD, but dopamine transporter (DAT) imaging with single-photon emission computed tomography (SPECT) or positron emission tomography (PET) tracers shows reduced presynaptic dopamine transport [78]. People with EOPD exhibit similar dopaminergic deficits to people with LOPD, though often with greater asymmetry [79]. Abnormal DAT imaging can rule out disorders like essential tremor or drug-induced parkinsonism [80]. DAT imaging provides useful adjunctive data but lacks the sensitivity and specificity to be used alone for diagnosis. Other diagnostic imaging techniques are actively being explored [78].

Emerging biomarker frameworks are shifting PD diagnosis toward a biological basis. The Synuclein, Neurodegeneration, and Genetics (SynNeurGe) model defines PD by the presence of misfolded synuclein in CSF or tissue, evidence of neurodegeneration on imaging, and genetic mutations [81, 82]. A different framework, the Neuronal alpha-Synuclein Disease Integrated Staging System (NSD-ISS), proposes reclassifying PD and Lewy body dementia under a shared biomarker-defined umbrella [83]. Though not yet in clinical use, these frameworks underscore the heterogeneity of PD and suggest a future in which CSF assays, imaging, and genomics complement traditional motor symptom-based diagnosis. Further research is needed to determine whether these tools perform differently in EOPD compared to LOPD.

## Treatment

Treatment requires special consideration due to its unique clinical profile, longer life expectancy, and increased risk of early treatment-related motor complications. While many studies in PD enrolled individuals over age 30, age-specific outcomes are rarely reported [84]. Similar to LOPD, a multidisciplinary approach is preferred to address the needs of people with EOPD effectively.

Levodopa combined with peripheral dopa decarboxylase inhibitors remains the gold standard treatment because of its symptomatic efficacy. In EOPD, its use has been debated given concerns over earlier onset of dyskinesias and motor fluctuations [85–88]. However, people with EOPD often require effective symptomatic therapy to maintain function, and relying on lower-potency agents can result in under-treatment and unnecessary disability. Importantly, multiple trials have shown that early use of levodopa improves quality of life without increasing long-term dyskinesia risk [88–91].

Dopamine agonists (DAs) are commonly used as initial therapy in younger people but require careful dose titration and regular monitoring due to increased susceptibility to impulse control disorders and sleep attacks [92]. Real-world prescribing patterns offer insights into the pharmacologic management of EOPD. In a study from Japan, dopamine agonists were the most frequently prescribed and often served as initial therapy; levodopa was less commonly used at diagnosis [93]. A study from China demonstrated that combinations of levodopa with dopamine agonists were commonly used with disease progression, and levodopa equivalent daily dosages gradually increased over time [94].

Monoamine oxidase type B (MAO-B) inhibitors (e.g., selegiline, rasagiline, and safinamide) offer modest symptomatic benefit by inhibiting the breakdown of dopamine in the brain. These agents may be employed as monotherapy in the early stages or as adjuncts to other therapies as the disease progresses [95]. Catechol-O-methyltransferase (COMT) inhibitors (e.g., entacapone, tolcapone, and the relatively newer opicapone) inhibit peripheral metabolism of levodopa and serve an adjunctive role [96]. However, similar to MAO-B inhibitors, evidence supporting their use comes largely from trials in heterogeneous PD populations, with limited age-specific data [97]. Amantadine has been repurposed for PD treatment and is typically introduced for management of dyskinesia [98, 99]. Anticholinergic agents, such as trihexyphenidyl and benztropine, were historically used for tremor management, but are limited by side effects including dry mouth, blurred vision, urinary retention, and confusion. Anticholinergic agents may be better tolerated in younger people with PD due to lower baseline cognitive vulnerability and thus may be useful in EOPD [100].

Infusion-based therapies, most notably levodopa-carbidopa intestinal gel (LCIG), are emerging options for advanced PD by improving the stability of dopaminergic delivery. LCIG is helpful in reducing motor fluctuations [101]. Apomorphine, a potent dopamine agonist with a rapid onset of action, can also be administered as continuous infusion [102]. However, both LCIG and apomorphine are typically reserved for advanced PD, and no trials have specifically examined their use in EOPD. Infusion therapies have also been evaluated in genetic forms of PD, where age of onset is often younger, and have shown benefit of motor complications. However, these findings are largely based on small cohorts or case series, and large-scale trials in EOPD are still lacking [103].

Treatment of non-motor symptoms generally mirrors LOPD. For EOPD, managing neuropsychiatric features, including anxiety, depression, and addictive behaviors such as gambling, hypersexuality, and substance use disorders should be prioritized [4, 104], especially since these issues may uniquely impact employment, disability, relationships, social stigma, and coping in EOPD.

While there are limited studies specifically evaluating exercise in EOPD, structured aerobic activity has substantial benefits for motor function, cognition, and quality of life in PD [105–107]. Given the greater physiological reserve and longer disease trajectory for EOPD, early and sustained engagement in exercise interventions can be particularly helpful for people with EOPD.

### Deep Brain Stimulation (DBS)

Deep brain stimulation (DBS) is a well-established treatment for advanced PD [108, 109]. Similar DBS outcomes are found in EOPD and LOPD, with improvements in motor impairment, depression, anxiety, impulsivity, quality of life, caregiver burden, and reductions in levodopa equivalent daily dose [110–112]. Although not specifically targeted to EOPD, the EARLYSTIM trial enrolled a younger population (mean age of 52) [113]. In this trial, DBS performed within three years of motor fluctuations/dyskinesia onset resulted with improved quality of life, motor function, and reduced levodopa need. Neuropsychiatric symptoms were stable or improved [114], which is particularly important given the higher prevalence of depression and suicidality in EOPD [20, 115–117]. Other studies evaluating the efficacy of DBS in the early stages of PD specifically excluded people with EOPD [118].

Limited data exist on DBS during pregnancy, a key consideration for women with EOPD. A case series of three women with EOPD due to *PRKN* mutations who underwent DBS before pregnancy reported no complications, and DBS settings did not need to be adjusted throughout pregnancy [119]. Further research and formal guidelines are needed for DBS and pregnancy [120].

The use of DBS in monogenic forms of EOPD remains an area of active investigation. Particular attention has been given to *GBA* [121]. While DBS is frequently considered, people with *GBA* have a higher risk of cognitive impairment [122] and may be prone to accelerated cognitive decline following DBS implantation. Subthalamic nucleus (STN) DBS is associated with faster cognitive decline and higher dementia risk for people with *GBA* [123, 124]. The retrospective nature of these studies means that results should be interpreted with caution, and careful consideration should be given to weighing improvements in motor function against possible detrimental effects on cognition in *GBA* for DBS. It also remains unknown whether globus pallidus interna (GPi) DBS could offer a more favorable cognitive profile in this population [125]. Generally favorable outcomes were demonstrated with DBS for PD with *PRKN* and *LRRK2* mutations, while *SNCA* mutation carriers may experience less robust motor improvements and more prominent cognitive decline after DBS [126].

## Conclusions

Majority of PD literature focuses on LOPD, and several features for EOPD and LOPD overlap. However, there are several important differences that should be considered to better diagnose and manage people with EOPD (Table 2). As biomarker and genetic assessments are conducted in more diverse populations, findings will undoubtedly provide better insight to pathogenesis and guide diagnostic and therapeutic advances. More research is still needed for clinical and therapeutic outcomes in EOPD. Differentiating EOPD from other likely treatable causes of parkinsonism in younger people, considering the monogenic variants more common in EOPD, investigating the experience of people with EOPD and validity of diagnostic tools and treatments particularly in EOPD remain priorities in clinic and research.

**Table 2:** Summary of differences between early- and late-onset parkinson’s disease (EOPD, LOPD)

Feature	EOPD	LOPD
Onset age	21–50	50+
Risk factors	Monogenic more common	Less common
Motor symptoms	Focal dystonia more common	Faster progression for motor impairment
Non-motor symptoms	Higher prevalence of depression	Higher prevalence of cognitive impairment and dementia
Diagnosis	Delayed diagnosis more common	Atypical neurodegenerative Parkinsonian disorders more common for differential diagnosis
Biomarkers	Sensitivity of synuclein biomarkers may be lower for monogenic forms	Co-pathologies more common
Treatment	Earlier and more frequent treatment-associated dyskinesias and motor fluctuations	More representation in treatment trials
Daily life impact	Higher risk for early retirement, unemployment Need for specific considerations for pregnancy and menstruation	Shorter disease duration due to older onset age More likely to impact postmenopausal women

## Key References

- Mehanna R, Marras C, Fleisher J, Post B, Kumar KR, Noyce A, et al. Diagnostic work up when suspecting early onset Parkinson disease (EOPD). Recommendations from the MDS EOPD study group. Parkinsonism Relat Disord. 2025;135:107852.
  - ○ MDS EOPD Study Group recommendations to prioritize brain MRI and laboratory investigation for Wilson’s disease in differential diagnosis for EOPD if no findings for secondary or genetic parkinsonism on history and exam.
- Wang J, Cheng N, Yao Z, Liu J, Kan X, Hui Z, et al. Early-onset Parkinson’s disease, regional and national burden, and its attributable risk factors in 204 countries and regions from 1990 to 2021: Results from the global burden of disease study 2021. Parkinsonism Relat Disord. 2025;134:107778.
  - ○ Global Burden of Disease Study 2021 data showing gender, age group, and regional differences for EOPD incidence and burden with projections for continued rise in burden and need for prompt diagnosis, equitable healthcare access, and tailored interventions.
- Milowska K, Mehanna R, Fleisher JE, Alcalay RN, Kumar KR, Marras C, et al. Unmet Need in Early-Onset Parkinson’s Disease: Deep Brain Stimulation and Pregnancy. J Park Dis. 2024;14:1277–82.
  - ○ A systematic review highlighting the lack of information on safety of DBS in pregnant women with EOPD and providing recommendations on how to support them.
- Das S, Ramteke H. A Comprehensive Review of the Role of Biomarkers in Early Diagnosis of Parkinson’s Disease. Cureus. 2024;16:e54337.
  - ○ A review of the biomarkers and their role in susceptibility, diagnosis, and prognosis underscoring the need for EOPD specific investigations

## Case

A 41-year-old man was referred to our movement disorders clinic for evaluation of gait difficulties by his primary care provider. His symptoms began at age 36, with subtle gait changes during running and a tremor in his left hand. Left-sided slowness gradually progressed, and he developed difficulty with fine motor tasks, such as typing, and “awkwardness” when walking or riding a bike. He also began having episodes of uncontrollable blinking and painful cramping in his left foot during exercise.

On examination, there was increased tone and bradykinesia in the left upper and lower extremities; a postural tremor in the left hand, without resting tremor; frequent blinking; and left foot posturing. He reported a history of anosmia and anxiety. Structural brain MRI was normal. DaTscan showed decreased radiotracer uptake in the right striatum. A hereditary PD and parkinsonism panel identified no mutations.

Carbidopa-levodopa therapy was initiated and provided significant improvement in motor symptoms, including left-foot dystonia, at 1.5 tablets (25/100 mg) three times daily. A diagnosis of EOPD was made.

Over the next two years, the patient developed symptoms on his right side and motor symptoms progressed, requiring increases in medication dosing and frequency. Botulinum toxin injections were initiated for blepharospasm and left lower extremity dystonia. He began experiencing motor fluctuations and left lower extremity dyskinesia and transitioned to longer-acting formulations of carbidopa/levodopa. At age 44, with increasingly disabling fluctuations and worsening dystonia, he was evaluated for deep brain stimulation (DBS). He proceeded with bilateral GPi DBS, with his goal of reducing both dystonia and dyskinesias. Postoperatively, he experienced marked improvement in dystonia, tone, and dyskinesias. We continued to gradually adjust the DBS settings over time.

### Lesson

Atypical features like postural tremor and focal dystonia in younger people can obscure the diagnosis of idiopathic PD. His dystonia-predominant phenotype also informed the therapeutic approach. The decision to pursue GPi-DBS – rather than STN-DBS – was guided by evidence supporting GPi’s efficacy in managing dystonia and levodopa-induced dyskinesias [127–129].

## Data Availability

No datasets were generated or analysed during the current study.
